# Roles of Elm1 in antifungal susceptibility and virulence in *Candida glabrata*

**DOI:** 10.1038/s41598-020-66620-7

**Published:** 2020-06-17

**Authors:** Yuya Ito, Taiga Miyazaki, Yutaka Tanaka, Takashi Suematsu, Hironobu Nakayama, Akihiro Morita, Tatsuro Hirayama, Masato Tashiro, Takahiro Takazono, Tomomi Saijo, Shintaro Shimamura, Kazuko Yamamoto, Yoshifumi Imamura, Koichi Izumikawa, Katsunori Yanagihara, Shigeru Kohno, Hiroshi Mukae

**Affiliations:** 1 Department of Respiratory Medicine, Nagasaki University Graduate School of Biomedical Sciences, Nagasaki, Japan; 2 Department of Respiratory Medicine, Nagasaki University Hospital, Nagasaki, Japan; 3 Department of Infectious Diseases, Nagasaki University Graduate School of Biomedical Sciences, Nagasaki, Japan; 4 Department of Infection and Host Defense, Tohoku Medical and Pharmaceutical University, Sendai, Japan; 5 Central Electron Microscope Laboratory, Nagasaki University Graduate School of Biomedical Sciences, Nagasaki, Japan; 6 Faculty of Pharmaceutical Sciences, Suzuka University of Medical Science, Suzuka, Japan; 7 Department of Laboratory Medicine, Nagasaki University Hospital, Nagasaki, Japan

**Keywords:** Microbiology, Fungi, Fungal pathogenesis

## Abstract

Elm1 is a serine/threonine kinase involved in multiple cellular functions, including cytokinesis, morphogenesis, and drug resistance in *Saccharomyces cerevisiae*; however, its roles in pathogenic fungi have not been reported. In this study, we created *ELM1*-deletion, *ELM1*-reconstituted, *ELM1*-overexpression, and *ELM1*-kinase-dead strains in the clinically important fungal pathogen *Candida glabrata* and investigated the roles of Elm1 in cell morphology, stress response, and virulence. The *elm1Δ* strain showed elongated morphology and a thicker cell wall, with analyses of cell-wall components revealing that this strain exhibited significantly increased chitin content relative to that in the wild-type and *ELM1*-overexpression strains. Although the *elm1Δ* strain exhibited slower growth than the other two strains, as well as increased sensitivity to high temperature and cell-wall-damaging agents, it showed increased virulence in a *Galleria mellonella-*infection model. Moreover, loss of Elm1 resulted in increased adhesion to agar plates and epithelial cells, which represent important virulence factors in *C. glabrata*. Furthermore, RNA sequencing revealed that expression levels of 30 adhesion-like genes were elevated in the *elm1Δ* strain. Importantly, all these functions were mediated by the kinase activity of Elm1. To our knowledge, this is the first report describing the functional characterization of Elm1 in pathogenic fungi.

## Introduction

Invasive candidiasis is among the most common fungal diseases in immunocompromised patients. Although about 40–50% cases of invasive candidiasis are caused by *Candida albicans*, the isolation rates of non-*albicans Candida* spp. have increased in the last decade^1,2^. *Candida glabrata* is the first or second most common cause of non-*albicans Candida* infections in various countries^2,3^. Increasing trend of *C. glabrata* infection is clinically important due to its intrinsically decreased susceptibility to azole antifungals^4–6^. Additionally, despite the limited numbers of therapeutic drugs, the emergence of multidrug-resistant *C. glabrata* isolates remains a serious problem in clinical practice^7^; therefore, the development of antifungal agents with a novel mechanism is urgently needed.

Calcineurin is a serine/threonine-specific protein phosphatase that exhibits various functions to control physiological processes, including morphogenesis, antifungal drug resistance, and virulence in pathogenic fungi^8^. The calcineurin signalling pathway has attracted attention as a novel target of antifungal therapy based on previous studies of pathogenic fungi, including *C. albicans*, *Cryptococcus neoformans*, and *Aspergillus fumigatus*^9–11^. In *C. glabrata*, the calcineurin signalling pathway regulates various functions through the downstream transcription factor Crz1; however, little is known about a Crz1-independent pathway^12,13^.

Elm1 is a serine/threonine protein kinase involved in multiple cellular functions, including cytokinesis, septin ring assembly, and morphogenesis in *Saccharomyces cerevisiae*^14–17^. In *S. cerevisiae*, Elm1 is regulated by calcineurin, with dephosphorylation of its C-terminus by calcineurin suppressing Elm1 activity^18^. Additionally, Elm1 phosphorylates Snf1, the yeast AMP-activated kinase (AMPK), with phosphorylated Snf1 co-regulating several genes with Crz1, including *HXT2* and *ENA1*^18^. Moreover, Elm1 also negatively regulates the Swe1 kinase by phosphorylation, with inhibition of Swe1 activity leading to activation of Cdc28, which regulates cell cycle^19–21^. A previous report on drug resistance revealed that mutations in several genes, including *CLA4*, *GIN4*, and *CDC28* functionally related to Elm1 increase sensitivity to cycloheximide by inhibiting the transcription of *PDR5* in *S. cerevisiae*^22^.

These findings indicate that *S. cerevisiae* Elm1 is regulated by calcineurin and exhibits various functions; however, its function and involvement in the virulence of the pathogenic fungus *C. glabrata* remain unknown. In this study, we elucidated the roles of Elm1 in stress response and virulence in the clinically important fungal pathogen *C. glabrata* by generating *elm1*Δ, *ELM1-*overexpression, and *ELM1-*kinase dead (KD) strains.

## Results

### Elm1 is involved in cell morphology and cell-wall structure in *C. glabrata*

**Figure 1:**
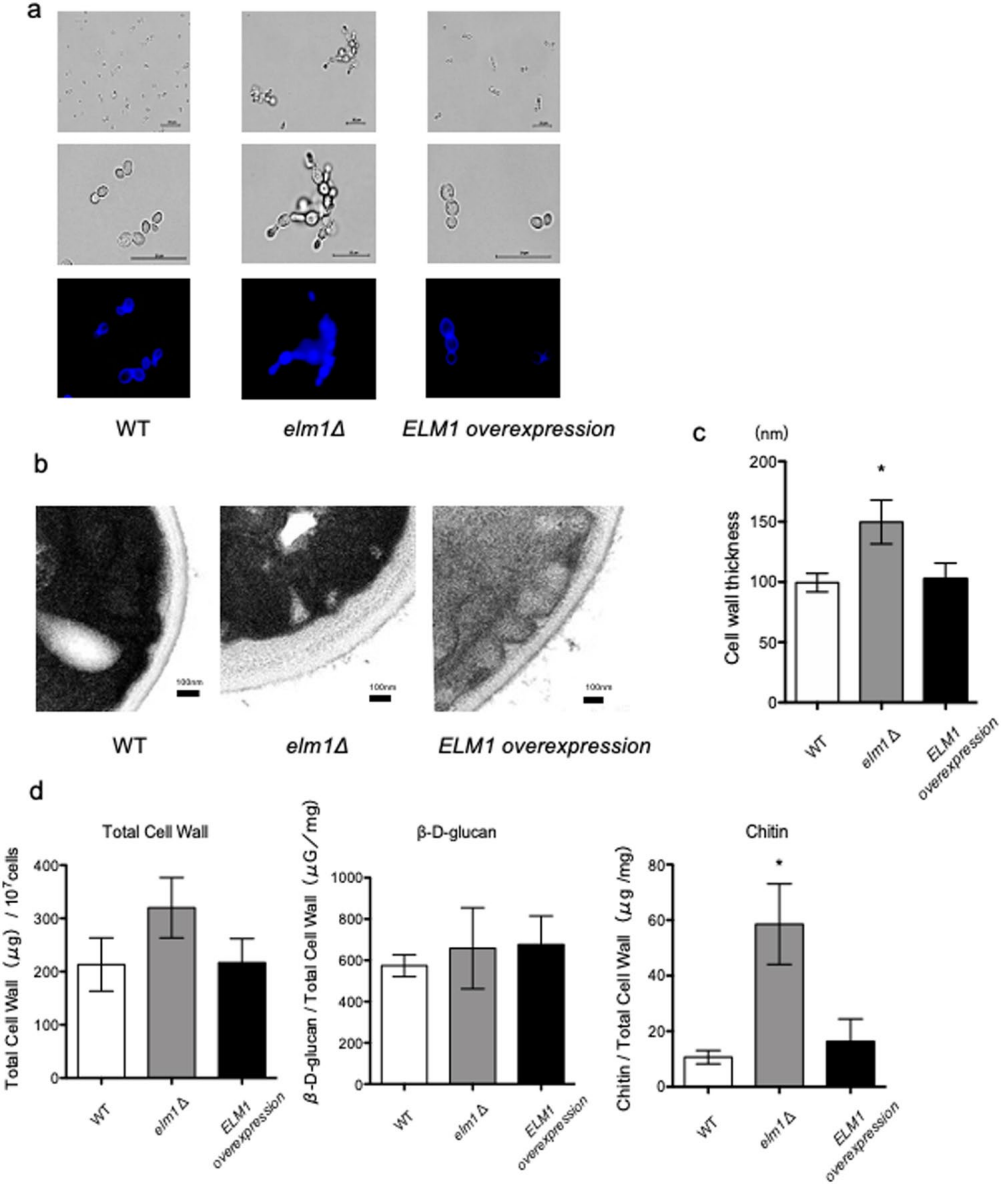
Cell morphology, cell-wall thickness, and cell-wall components. (**a**) Logarithmic phase *C. glabrata* cells grown in SC-trp medium at 30 °C were stained with Calcofluor white. Stained cells were observed by microscopy using bright-field and BZ-X filter for DAPI. *C. glabrata* strains: WT, TG11; *elm1*Δ, TG352; and *ELM1*-overexpression, TG353. Scale bars, 20 μm. (**b**,**c**) Logarithmic phase *C. glabrata* cells were observed by TEM. Scale bars, 100 nm. Cell-wall thickness was determined by measuring the thickest site in 50 randomly selected cells. *P < 0.0001, one-way analysis of variance. (**d**) Measurement of total cell-wall, β-D-glucan, and chitin contents in *C. glabrata* cells. Data represent the results of at least three independent experiments. Error bars represent standard deviations. *P = 0.0018, one-way analysis of variance.

Elm1 is associated with an elongated morphology, pseudohyphal growth, and cell cycle progression in *S. cerevisiae*^17^. To examine the involvement of Elm1 in cell morphology in *C. glabrata*, we observed wild-type, *elm1Δ*, and *ELM1-*overexpression strains using a fluorescence microscope and transmission electron microscope (TEM). Similar to *S. cerevisiae*, the *elm1Δ* strain exhibited an elongated morphology in *C. glabrata*, which was restored to the yeast form by reintroduction of an intact *ELM1* gene into the mutant (Fig. 1a). Additionally, the *elm1Δ* strain showed a strong fluorescence intensity as a whole in Calcofluor white staining (Fig. 1a) and had a significantly thicker cell wall and higher total cell-wall content per cell relative to the wild-type and *ELM1*-overexpression strains (Fig. 1b–d). Moreover, analysis of cell-wall components revealed that, although there was no difference in the β-D-glucan content, the *elm1Δ* strain showed significantly increased chitin content as compared with the wild-type and *ELM1*-overexpression strains (Fig. 1d).

### Effects of *ELM1* deletion on cell growth and cell-wall integrity

**Figure 2:**
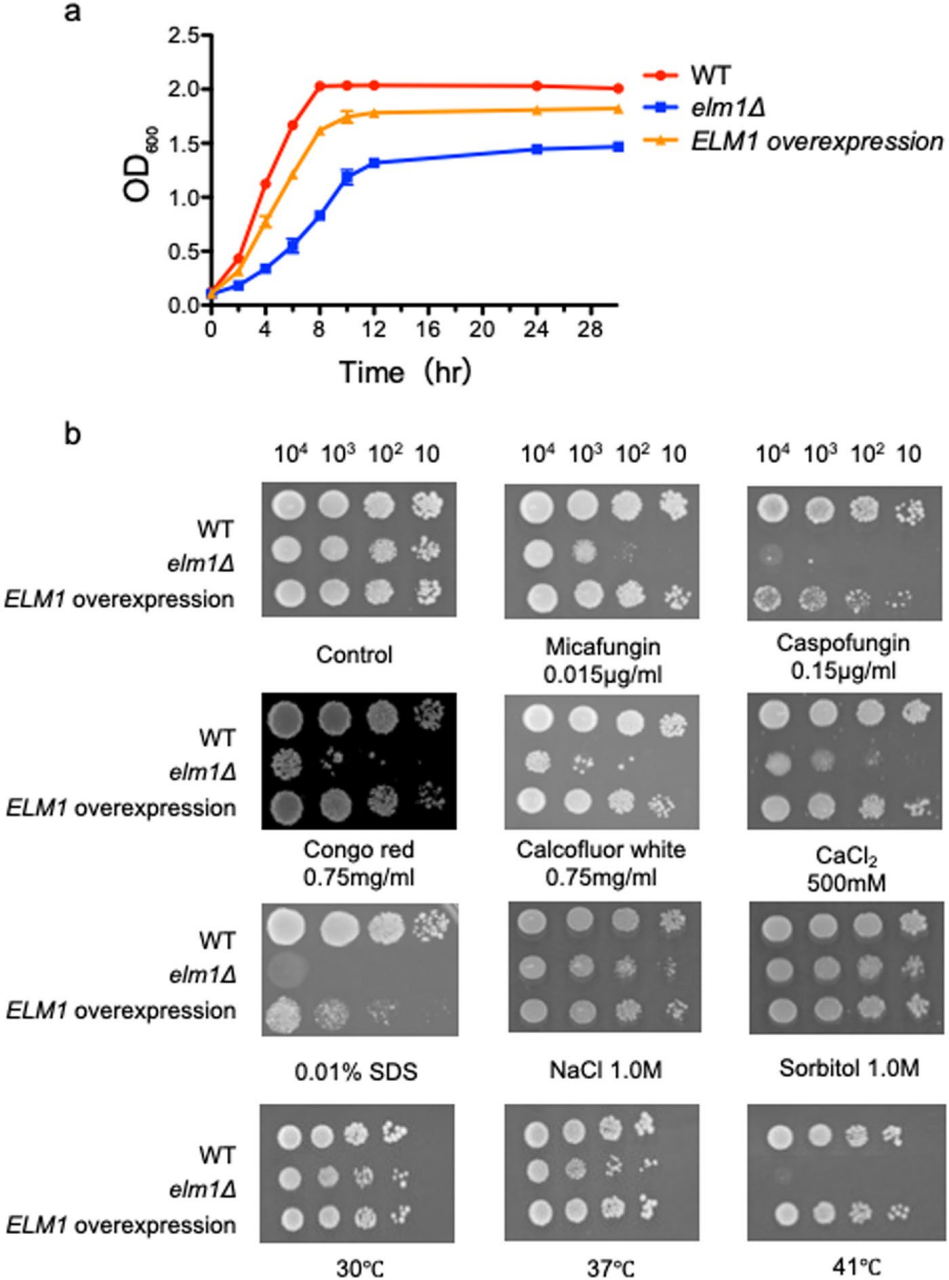
Growth curves and spot dilution assay. (**a**) Logarithmic phase *C. glabrata* cells grown in SC-trp medium at 37 °C were washed twice with dH_2_O, diluted to an OD_600_ of 0.1 with fresh SC-trp medium and incubated at 37 °C with shaking at 200 rpm. The OD_600_ of cultures was measured at 2, 4, 6, 8, 10, 12, 24 and 30 h. *C. glabrata* strains: WT, TG11; *elm1*Δ, TG352; and *ELM1*-overexpression, TG353. Error bars represent standard deviations. The average doubling times per strain were obtained from three independent experiments: wild-type, 1.24 h; *elm1Δ*, 3.09 h; and *ELM1*-overexpression, 1.42 h. (**b**) Serial 10-fold dilutions of logarithmic-phase cells of *C*. *glabrata* cells were spotted onto SC-trp agar plates containing the indicated compounds at the specified concentrations, incubated at 30 °C (unless otherwise specified) for 48 h, and photographed. Images are representative of three independent replicate experiments. SDS; sodium dodecyl sulfate.

The growth capacity of the *elm1*Δ cells was examined in SC-trp broth at 37 °C. The *elm1Δ* strain showed slower growth and a 2-fold longer doubling time than the other two strains (Fig. 2a). We then examined the sensitivity to cell-wall-damaging agents using microdilution and spot dilution assays. The *elm1Δ* strain showed increased susceptibility to micafungin, caspofungin and amphotericin B as compared with the wild-type and *ELM1*-overexpression strains in broth microdilution assays (Table 1). In spot dilution assays, the *elm1Δ* strain showed increased sensitivity to high temperature and cell-wall-damaging agents, including echinocandins, Congo red, Calcofluor white, sodium dodecyl sulphate (SDS), and calcium chloride (Fig. 2b). On the other hand, the *elm1Δ* strain showed similar resistance to osmotic stress, such as sodium chloride and sorbitol, as the wild-type strain. These results suggested that Elm1 is required for cell-wall integrity in *C. glabrata*.

**Table 1:** MICs of *C. glabrata* strains.

Strain	MIC (μg/mL)				
	Anidulafungin	Micafungin	Caspofungin	Voriconazole	Amphotericin B
WT (TG11)	0.12	0.03	0.12	0.25	0.5
Δ*elm1* (TG352)	0.12	0.015	0.06	0.25	0.25
*ELM1* overexpression (TG353)	0.12	0.03	0.12	0.25	0.5

### Loss of Elm1 results in increased cell adhesion and leads to hypervirulence

**Figure 3:**
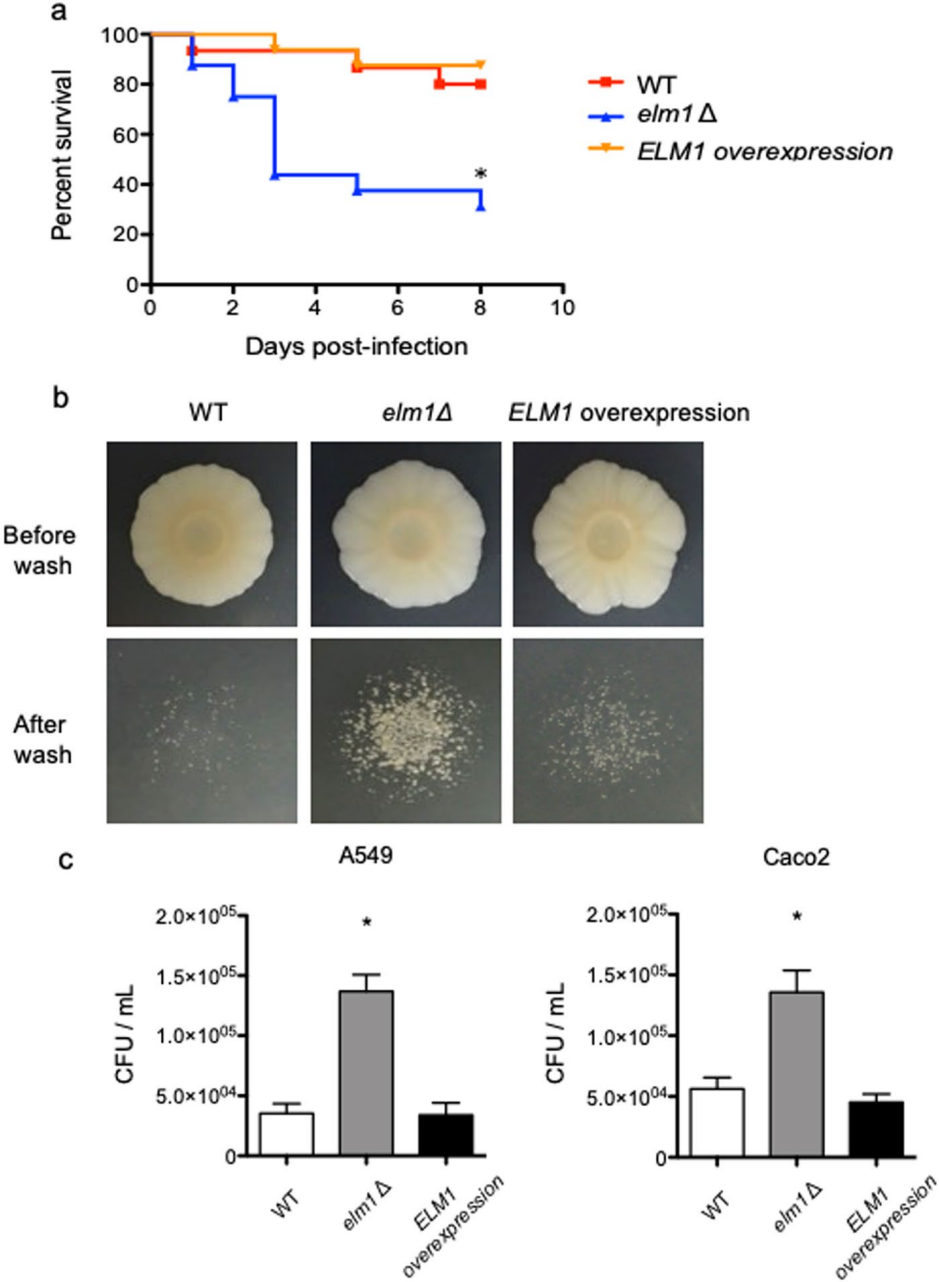
Virulence assay and adhesion assays. (**a**) Groups of 16 healthy larvae were inoculated with 10 μL of *C. glabrata* cell suspensions (1.0 × 10^8^ cells/mL) into the haemocoel and incubated in the dark at 37 °C, and survival was monitored daily for 7 days. *C. glabrata* strains: WT, TG11; *elm1*Δ, TG352; and *ELM1*-overexpression, TG353. Kaplan–Meier curves were generated and compared by the log rank (Mantel–Cox) test using GraphPad Prism 5 software (GraphPad Software, La Jolla, CA) (https://www.graphpad.com/scientific-software/prism/). The *elm1Δ* strain was significantly more virulent than the wild-type and *ELM1*-overexpression strains. *P < 0.01: *P* = 0.006 for e*lm1*Δ vs. wild-type, *P* = 0.0009 for *elm1*Δ vs. *ELM1* overexpression, and *P* = 0.5870 for wild-type vs. *ELM1* overexpression. (**b**) Logarithmic phase *C. glabrata* cells were grown in SC-trp medium at 37 °C, washed twice with dH_2_O, adjusted to 1.0 × 10^7^ cells/mL, and 5 μL of the cell suspension was spotted onto SC-trp agar plate and incubated at 37 °C for 10 days. Colonies were photographed before and after washing with dH_2_O. (**c**) Logarithmic phase A549 and Caco2 cells were seeded in 24-well plates at a density of 1.0 × 10^5^ cells/well and grown to confluence. Logarithmic phase *C. glabrata* cells grown in SC-trp medium at 37 °C were washed twice with PBS, adjusted to 3.0 × 10^5^ cells/mL, and 1 mL of the cell suspension was added to epithelial cell monolayers. The co-cultures were incubated at 37 °C in a humid atmosphere with 5% CO_2_ for 30 min. Planktonic cells were removed by washing twice with PBS, and epithelial cells monolayers were lysed using 0.1% Triton-X. Adhered cells were recovered and plated on YPD agar plates for CFU. *P < 0.0001, one-way analysis of variance.

The effect of *ELM1* deletion on virulence was first examined using a mouse model of disseminated candidiasis. Immunocompetent mice infected with the *elm1Δ* strain showed slightly reduced fungal burden in the kidney and spleen as compared with those infected with the wild-type and *ELM1*-overexpression strains (see Supplementary Fig. S1). However, the mice infected with the *elm1Δ* strain exhibited significantly increased fungal burden in the lung as compared with those infected with the other two strains. In lung histopathology, fungal embolization of the pulmonary artery was observed in mice infected with the *elm1Δ* strain but not in mice infected with the wild-type strain. Therefore, it was difficult to evaluate virulence of the *elm1*Δ strain using the mouse model of disseminated candidiasis. We then conducted a virulence assay using a *Galleria mellonella*-infection model. In this model, the *elm1Δ* strain was significantly more virulent than the wild-type and *ELM1*-overexpression strains (Fig. 3a). Because adhesion is known as a key virulence factor of *C. glabrata*, we performed adhesion assays using agar plates and epithelial cells. On SC-trp agar plates, the *elm1Δ* strain exhibited enhanced adhesion as compared with the wild-type and *ELM1*-overexpression strains (Fig. 3b). Moreover, the *elm1Δ* strain showed significantly higher adhesion to epithelial cells (A549 and Caco2 cells) as compared with the wild-type and *ELM1*-overexpression strains (Fig. 3c). These results suggested that loss of Elm1 induced increased adhesion, which could lead to hypervirulence as observed in the *Galleria mellonella*-infection model.

### Expression profiling of Elm1-mediated genes in *C. glabrata*

**Figure 4:**
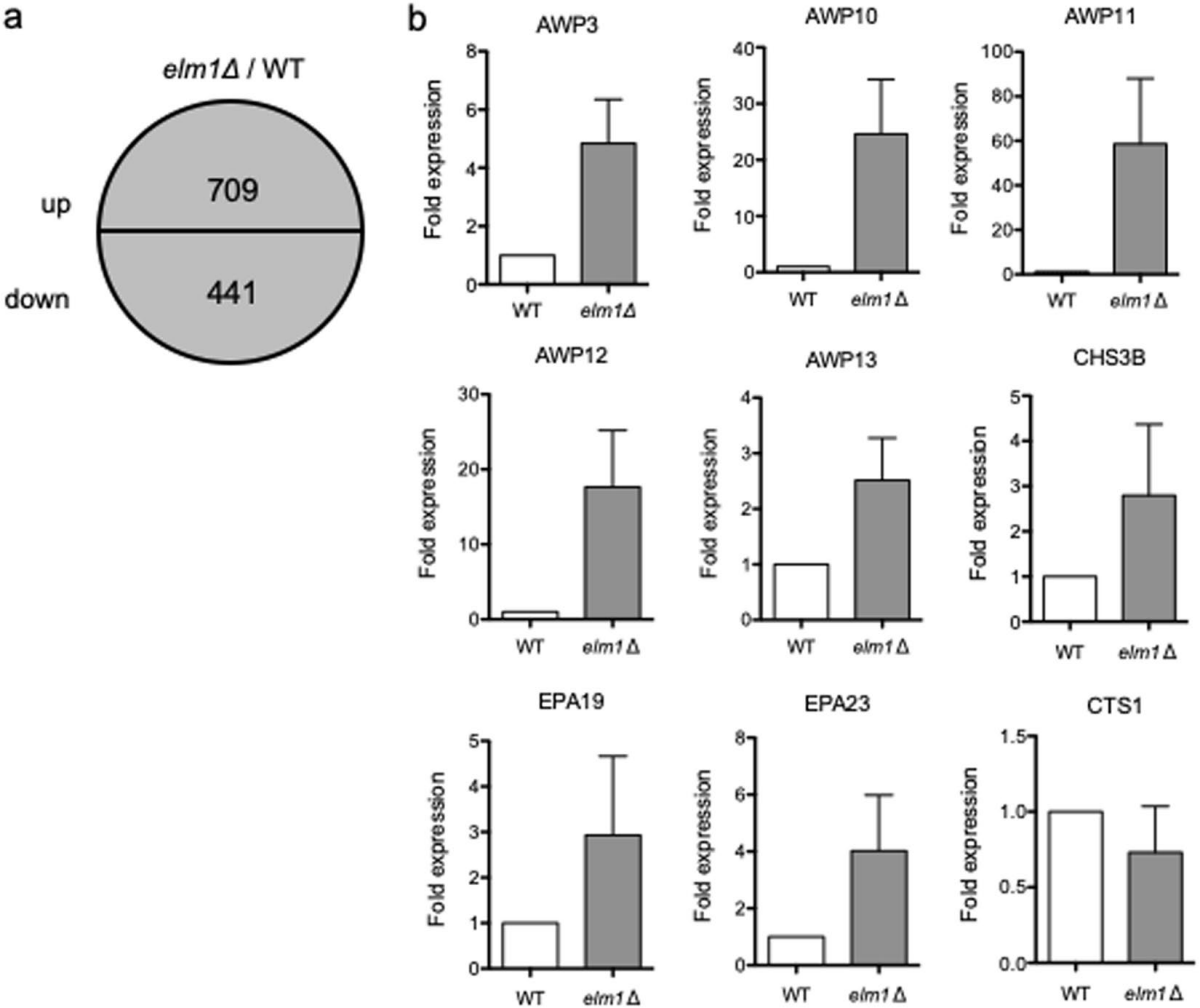
Identification of genes regulated by Elm1. (**a**) Expression levels of total 1150 genes altered by >2-fold in the *elm1*Δ strain relative to the wild-type strain (709 genes were upregulated, and 441 genes were downregulated). *C. glabrata* strains: WT, TG11; and *elm1*Δ, TG352. (**b**) qRT-PCR analysis of the upregulated and downregulated genes. Results are presented as fold expression relative to levels in the wild-type strain. The means and standard deviations of three independent experiments are shown.

To investigate the transcriptional profile of the *elm1*Δ strain, genome-wide expression analyses were conducted using RNA-sequencing (RNA-seq). Expression levels of 1150 genes were altered by >2-fold in the *elm1*Δ strain, of which 709 genes were upregulated and 441 genes were downregulated relative to the wild-type strain (Fig. 4a). Gene name/ID and related information were provided for the upregulated and downregulated genes in Supplementary Table S1–S4. Among 1150 genes, those related to adhesion or chitin were selected using Candida Genome Database (http://www.candidagenome.org/). Among genes upregulated in the *elm1Δ* strain, there were 30 encoding adhesin-like glycophosphatidylinositol (GPI) proteins, including those from the *EPA* family (*EPA12*, *EPA19*, *EPA20*, and *EPA23*) and the *AWP* family (*AWP1*, *AWP3*, *AWP6*, and *AWP9~13*) (Table 2). By contrast, six adhesin-related genes were downregulated in the *elm1Δ* strain. Among genes involved in the chitin biosynthesis, *CHS3B* encoding a class IV chitin synthase was upregulated, whereas *CTS1* encoding an endochitinase involved in cell separation was downregulated in the *elm1Δ* strain. To confirm RNA-seq results, we performed qRT-PCR to analyse the seven upregulated genes and one downregulated gene, revealing generally consistent results with those from RNA-seq analysis (Fig. 4b).

**Table 2:** Altered expression levels of adhesin-related and chitin-related genes in the *C. glabrata elm1Δ* strain.

Systematic name and function	Gene	Fold change in expression	*S. cerevisiae* ortholog
Upregulated adhesin-related gene			
CAGL0M00110g	*AWP11*	7.2	
CAGL0J00110g		6.6	
CAGL0I11011g		6.5	
CAGL0L00157g		5.5	
CAGL0H00110g		4.8	
CAGL0I00220g	*EPA23*	4.8	*FLO1*
CAGL0F09273g		4.2	
CAGL0I00110g		4.2	
CAGL0E00110g		3.9	
CAGL0J11891g	*AWP3*	3.9	
CAGL0A00110g	*EPA19*	3.6	*FLO1*
CAGL0H10626g	*AWP13*	3.6	
CAGL0G10219g	*AWP12*	3.5	
CAGL0J01800g		3.5	
CAGL0H00132g		3.4	
CAGL0F00110g	*AWP10*	3.3	
CAGL0G10175g	*AWP6*	2.8	*DAN1*
CAGL0K13002g	*AED2*	2.8	
CAGL0C03575g		2.5	*AGA1/YNR044W*
CAGL0J02530g		2.5	
CAGL0A04873g		1.9	
CAGL0E00275g	*EPA20*	1.9	FLO5
CAGL0B05093g	*AWP9*	1.7	
CAGL0I10098g	*PWP7*	1.6	*FLO5*
CAGL0K10164g		1.5	*SED1/YDR077W*
CAGL0K13024g	*AED1*	1.4	
CAGL0G04125g		1.2	*SAG1*
CAGL0J00253g		1.1	*MTL1/YGR023W*
CAGL0J02508g	*AWP1*	1.1	
CAGL0M00132g	*EPA12*	1	*FLO1*
Downregulated adhesin-related gene			
CAGL0L13332g	*EPA13*	−3.8	*FLO9*
CAGL0B00110g	*AWP8*	−2.7	
CAGL0E02915g		−2.3	*SCW11/YGL028C*
CAGL0I10340g	*PWP5*	−1.3	*FLO1*
CAGL0J04950g	*EPA21*	−1.2	*FLO1*
CAGL0L04268g	*EPA11*	−1.1	*FLO9*
Upregulated chitin-related gene			
CAGL0I04840g	*CHS3B*	1.6	*CHS3*
Downregulated chitin-related gene			
CAGL0M09779g	*CTS1*	−3.3	*CTS1*

### Phenotypes of the *ELM1*-reconstituted strain and *ELM1*-KD strain in *C. glabrata*

We also created an *ELM1*-reconstituted strain in which *ELM1* was expressed under its native promoter. The phenotypes of this strain were similar to those of the wild-type and *ELM1*-overexpression strains in cell morphology, *in vitro* growth, and stress responses (see Supplementary Fig. S2).

In *S. cerevisiae*, the kinase domain of Elm1 is encoded by *ELM1 c.255–267*. Because these putative amino acid sequences are highly conserved in *C. glabrata ELM1* (*CgELM1 c.265–277*), we constructed an *ELM1*-KD strain lacking the corresponding region in *C. glabrata* (see Supplementary Fig. S3). The *ELM1-*KD strain was confirmed by separation of phosphorylated and dephosphorylated forms of Elm1 by western blotting using Phos-tag SDS-PAGE (see Supplementary Fig. S4). The phenotypes of the *ELM1-*KD strain were similar to those of the *elm1Δ* strain in cell morphology, *in vitro* growth, and stress responses (see Supplementary Fig. S2). In addition, the expression levels of genes involved in adhesion and chitin synthesis in the *ELM1-*KD strain were also similar to those in the *elm1Δ* strain (see Supplementary Table S5). These results suggested that the *elm1Δ* phenotypes observed in this study, including elongated cell morphology, increased sensitivity to cell-wall-damaging agents, and increased expression of genes involved in adhesion and chitin synthesis, were due to loss of the Elm1 kinase activity in *C. glabrata*.

## Discussion

To the best of our knowledge, this represents the first report on the biological functions of Elm1 in pathogenic fungi to date. In this study, we demonstrated that *C. glabrata* Elm1 was involved in cell morphology, cell-wall integrity, adhesion, and virulence, and that these functions were dependent on the Elm1 kinase activity.

Previous studies in *S. cerevisiae* have shown that Elm1 is involved in the spindle position checkpoint and morphogenesis check point during cell division, and that loss of Elm1 causes a delay in cytokinesis and leads to elongated morphology and slow growth^14,15,23^. In the present study, loss of Elm1 in *C. glabrata* exhibited a similar elongated morphology and slow growth, presumably associated with the same mechanisms as those in *S. cerevisiae*. Additionally, RNA-seq analysis of the *elm1Δ* strain indicated downregulation of genes related to post-mitotic cell separation, including *CTS1*, *EGT2, DSE1, DSE2, DSE3*, and *SCW11*; therefore, it is likely that the elongated morphology of this strain resulted from a defect in cell separation after mitosis^24^. In fact, loss of *C. glabrata Ace2*, which regulates these genes, exhibits a clumpy growth phenotype^25^.

Mutations in genes associated with the cell cycle, such as *cdc3*, *cdc4*, *cdc7*, *cdc24*, and *cdc28*, result in increased chitin content and aberrant distribution of chitin in the cell wall in *S. cerevisiae*^26^. This delocalization of chitin deposition is presumably caused by a lack of normally regulated directional apparatus^26^. In the present study, the *elm1Δ* strain displayed a significantly thicker cell wall and increased chitin content. Elm1 is involved in the cell cycle by activating Cdc28 via the Swe1 kinase in *S. cerevisiae*^19–21^. Because *C. glabrata* possesses orthologues of these genes, loss of Elm1 might cause a phenotype similar to Cdc28 mutation. Additionally, the *elm1Δ* strain showed upregulation of *CHS3B* and downregulation of *CTS1* according to RNA-seq analysis. In *S. cerevisiae*, chitin synthase 3 (Chs3), a homologue of *C. glabrata* CHS3B, is required for the synthesis of cell-wall chitin and the chitin ring during bud emergence^27^. Although it is unclear how much CHS3B is involved in the chitin synthesis in *C. glabrata*, RNA-seq results suggest that imbalance between chitin synthesis and chitin degradation affects the chitin content in the cell wall.

Generally, increased chitin content in the cell wall is thought to increase sensitivity to Congo red and Calcofluor white but decrease susceptibility to echinocandin drugs^28–30^. However, the *C. glabrata elm1Δ* strain exhibited increased sensitivity to all cell-wall-damaging agents examined, including echinocandins, despite the increased chitin content in the cell wall. The slower growth of the *elm1Δ* strain may have a potential impact on sensitivity to environmental stressors when examined using spot dilution assays. In *S. cerevisiae*, mutations in *cak1*^*P212S*^ and *cla4*, which are involved in cell cycle progression, cause hyperpolarized growth and cell-wall vulnerability similar to that observed in the *elm1Δ* strain^31^. These phenotypes are due to hyperpolarized secretion of glucan synthase and lack of reinforcement of the lateral cell walls^31^. Moreover, mutation of *SNF1*, a gene partially regulated by Elm1, reduces cell- wall strength in *S. cerevisiae*, suggesting that carbon metabolism might affect cell-wall integrity^32^. In the present study, although cell-wall β-D-glucan content in the *elm1Δ* strain was similar to that in the wild-type strain, RNA-seq analysis revealed the downregulation of genes related to glycogen synthesis and carbohydrate metabolism in the *elm1Δ* strain. These results suggest that the quality of β-D-glucan is also important for the cell-wall integrity, and that its defect might explain the increased susceptibility of the *elm1Δ* strain to inhibitors of β-D-glucan synthesis (echinocandins).

Despite the vulnerability of cell-wall integrity, the *C. glabrata elm1Δ* strain showed increased virulence in the *G. mellonella*-infection model. In *C. glabrata*, adhesion is the first step to infection of the host, with adhesion to host tissue or medical devices the most important virulence factor^33,34^. Previous genome-wide in silico inspection revealed that *C. glabrata* harbours 67 of genes encoding adhesin-like GPI proteins, including those from the *EPA* family, *PWP* family, and *AWP* family^35^. These adhesin-like GPI proteins play important roles in virulence^36,37^. In our study, the *C. glabrata elm1Δ* strain showed increased adhesion to both agar plates and epithelial cells. In addition, 30 genes encoding adhesin-like GPI protein, including *EPA* family (*EPA12*, *EPA19*, *EPA20*, and *EPA23*) and *AWP* family (*AWP1*, *AWP3*, *AWP6*, and *AWP9~13*), were upregulated in the *elm1Δ* strain. Therefore, our results suggested that hypervirulence of the *elm1*Δ strain was attributed at least in part to enhanced adhesion.

In *S. cerevisiae*, the Elm1 kinase activity is important for the maintenance of cell morphology and cell cycle progression^14–16^. We found that the morphology, drug sensitivity, and the expression levels of genes related to adhesion and chitin synthesis observed in the *ELM1*-KD strain were similar to those in the *elm1*Δ strain, suggesting that loss of the kinase activity was responsible for the *elm1*Δ phenotypes in *C. glabrata*.

In conclusion, this is the first report demonstrating the effects of Elm1 on cell morphology, stress response, and virulence in *C. glabrata*. Loss of Elm1 increased cell vulnerability, but increased adhesion, resulting in increased virulence *in vivo* and suggesting the possibility of another kind of stress response in pathogenic fungi. The overexpression of *ELM1* in the *elm1Δ* strain recovered the phenotypes to the wild-type levels but did not induce additional effects. The fungal cell walls are currently considered as ideal drug targets and also primarily recognized by host immune systems. Understanding the specific stress-response mechanisms of pathogenic fungi is important for future drug development.

## Materials and Methods

### Strains, media, cell lines and compounds

The *C. glabrata* strains, primers and plasmids used in this study are listed in Supplementary Tables S6, S7 and S8, respectively. Detailed information on materials (media, cell lines, and compounds) and methods for plasmid and strain construction is presented in the supplementary information file (see Supplementary materials and methods online).

### Microscopy and measurement of cell wall thickness

Logarithmic phase *C. glabrata* cells grown in SC-trp medium at 30 °C were observed by bright-field using a BZ-X700 microscope (Keyence, Osaka, Japan). To visualize cell-wall chitin, logarithmic phase *C. glabrata* cells were stained by incubation for 15 min in 100 ng/mL Calcofluor white and observed using a BZ-X filter for 4’,6-diamidino-2-phenylindole (DAPI). To evaluate cell-wall thickness, *C. glabrata* cells were observed by TEM. Briefly, logarithmic phase *C. glabrata* cells grown in SC-trp medium at 30 °C were washed twice with dH_2_O and adjusted to 1 × 10^7^ cells/mL. The cells were then fixed in 4% glutaraldehyde in 0.1 M phosphate buffer at 4 °C for 3 days, washed four times in the same buffer, post-fixed in 1% potassium permanganate in ultrapure water at 4 °C for 2 h, and washed four times with ultrapure water. The cells were then dehydrated in an acetone series (50%, 70%, 80%, 90%, 95%, 99.5%, and 100%), substituted in propylene oxide, and embedded in epoxy resin. Ultra-thin sections (80 nm) were then stained with uranyl acetate, followed by lead citrate, and the specimens were observed by TEM (JEM-1200EX; JEOL). Cell-wall thickness was determined by measuring the thickest site in 50 randomly selected cells.

### Analysis of cell wall composition

Cell-wall sugar composition was measured as described previously with some modification^38^. Logarithmic phase *C. glabrata* cells were grown in SC-trp medium harvested by centrifugation, washed three times with deionized water, and extracted with 0.1 M NaOH at 95 °C for 90 min. The alkali-insoluble pellet was washed twice with deionized water and lyophilized. Cell-wall β-glucan content in the alkali-insoluble fraction was determined as described previously with slight modification^39^. The lyophilized alkali-insoluble fraction (2 mg) was suspended in 10 mM Tris-HCl (pH 7.4) containing 1 mg/mL Zymolyase 100 T (Nacalai Tesque, Tokyo, Japan) and incubated at 37 °C for 24 h. The precipitate was collected by centrifugation at 10,000 × *g* for 10 min, and half of the obtained supernatant was dialyzed overnight against deionized water. The hexose content of the pellet and of the dialyzed and un-dialyzed portions of the supernatant was determined using the phenol-H_2_SO_4_ method. Cell-wall chitin content in the fraction was determined as described by Roncero and Duran with some modifications^40^. The lyophilized alkali-insoluble fraction (50 mg) was hydrolysed with 1 M H_2_SO_4_ at 100 °C for 4 h and then neutralized, after which the precipitate was separated by centrifugation, and the supernatant was dried by evaporation and dissolved in 2 mL of deionized water. We then added 1 mL of acetylacetone solution [10% (v/v) acetylacetone in 1.25 M sodium carbonate] to 500 μL of this solution and incubated the mixture at 90 °C for 1 h. We then added 10 mL of 99.5% ethanol and 1 mL of Ehrlich’s reagent [1% 4-dimethylaminobenzaldehyde, 18% (v/v) hydrogen chloride, and 50% (v/v) ethanol] to the mixture, and the content of chitin was measured by absorbance at 530 nm as glucosamine content.

### Growth curve and doubling time measurement

Logarithmic phase *C. glabrata* cells grown in SC-trp medium at 37 °C were washed twice with dH_2_O and diluted to an optical density at 600 nm (OD_600_) of 0.1 with fresh SC-trp medium. The diluted cells were incubated at 37 °C with shaking at 200 rpm, and the OD_600_ of the cultures was measured at 2, 4, 6, 8, 10, 12, 24, and 30 h. The doubling time were calculated as previously described^41^. The averages of the doubling times were obtained from three independent experiments.

### Drug-susceptibility assay

A spot dilution assay was performed as described previously^42^. Briefly, logarithmic phase cells grown in SC-trp medium were adjusted to 2 × 10^6^ cells/mL, and 5 μL of serial 10-fold dilutions were spotted onto SC-trp agar plates containing each compound at the indicated concentrations. Plates were incubated at 30 °C for 48 h, unless otherwise indicated. Antifungal susceptibility testing was performed using a commercially prepared colorimetric panel, Sensititre Yeast One microtiter panel (TREK Diagnostic Systems, Ltd, East Grinstead, UK) according to the manufacturer’s instructions. This test was also performed with the *C. glabrata* strains adjusted to 6.0 × 10^6^ cells/mL (corresponding to an OD_530_ of 0.1). All assays were performed at least three times on independent occasions.

### Adhesion assays

Adhesion to agar plates was determined by modifying the previously described methods^43,44^. Logarithmic phase cells grown in SC-trp medium at 37 °C were washed twice with dH_2_O, adjusted to 1.0 × 10^7^ cells/mL, and 5 μL of the cell suspension was spotted onto SC-trp agar plates for incubation at 37 °C for 10 days. Adhered cells were removed by washing with dH_2_O, and colonies were photographed before and after removal from the agar surface.

Adhesion to epithelial cells was determined as described previously^45^. Logarithmic phase epithelial cells were seeded in 24-well plates at a density of 1.0 × 10^5^ cells/well in 1 mL of DMEM and grown to confluence at 37 °C in a humid atmosphere with 5% CO_2_. Logarithmic phase *C. glabrata* cells grown in SC-trp medium at 37 °C were washed twice with phosphate-buffered saline (PBS), adjusted to 3.0 × 10^5^ cells/mL in PBS, and 1 mL of the cell suspension was added to epithelial cell monolayers. The plate with the co-cultures was centrifuged at 200 × *g* for 1 min and incubated at 37 °C in a humid atmosphere with 5% CO_2_ for 30 min. After adhesion, planktonic cells were removed by washing twice with PBS, and epithelial cells monolayers were lysed by using 0.1% Triton-X. Adhered cells were recovered and plated on YPD agar plates for quantification of CFU. This assay was performed at least three times on independent occasions to ensure reproducibility.

### Ethics statement and virulence assays

All animal experiments were performed in accordance with the Guide for the Care and Use of Laboratory Animals^46^ and all institutional regulations and guidelines for animal experimentation following review and approval by the Institutional Animal Care and Use Committee of Nagasaki University (approval number 1906121536-3). Mouse experiments were performed as described previously^47^. Detailed information on materials and methods for mouse experiments and histopathological examination is presented in the supplementary file.

To examine the mortality of *G. mellonella* infected with *C. glabrata*, groups of 16 healthy larvae (approximately 300 mg) were inoculated with 10 μL of *C. glabrata* cell suspensions (1.0 × 10^8^ cells/mL) into the haemocoel using a Hamilton syringe through the last left pro-leg^48^. Inoculated larvae were incubated in the dark at 37 °C, and survival was monitored daily for 7 days. Sixteen larvae inoculated with PBS were used as controls and no larvae dead. Survival was plotted on a Kaplan-Meier curve for each *C. glabrata* strain, and the log rank (Mantel-Cox) test was used for pairwise comparison of percent survival using GraphPad Prism 5 software (GraphPad Software, La Jolla, CA) (https://www.graphpad.com/scientific-software/prism/). A *P* < 0.05 was considered statistically significant.

### RNA-seq

RNA-seq library preparation, sequencing, mapping, and gene expression analysis were performed by DNAFORM (Yokohama, Japan). Briefly, RNA quality was assessed by Bioanalyzer (Agilent Technologies, Santa Clara, CA) to ensure an RNA integrity number >7.0, and A260/280 and 260/230 ratios >1.8. Total RNA was purified using a Magnosphere UltraPure mRNA Purification Kit (Takara Bio Inc.). Libraries prepared using a SMARTer Stranded Total RNA-Seq Kit (Takara Bio Inc.) were processed using a HiSeq system as 150-bp paired-ends (Illumina Inc., San Diego, CA). The quality of RNA-seq results were assessed using FastQC (v.0.11.5; https://www.bioinformatics.babraham.ac.uk/projects/fastqc/), and the raw reads were trimmed and quality filtered with Trim Galore! (v.0.4.4; https://www.bioinformatics.babraham.ac.uk/projects/trim_galore/), Trimmomatic (v.0.36; http://www.usadellab.org/cms/?page=trimmomatic), and Cutadapt (v.1.9.1; https://cutadapt.readthedocs.io/en/stable/index.html) software^49,50^. Trimmed and filtered reads were aligned against the *C. glabrata* CBS138 reference genome using STAR (v.2.4.2a)^51^. After counting reads on gene features using the featureCounts tool (v.1.5.0-p2)^52^, quantitative differential expression analysis between different conditions was performed using DESeq. 2 (v.1.20.0; https://bioconductor.org/packages/release/bioc/html/DESeq. 2.html). Enrichment analysis for Gene Ontology (GO) was performed using clusterProfiler (v.3.6.0; https://bioconductor.org/packages/release/bioc/html/clusterProfiler.html). An annotation package of *Candida glabrata* for GO analysis was prepared using AnnotationForge (v.1.22.2; https://bioconductor.org/packages/release/bioc/html/AnnotationForge.html) and the GO annotation file obtained at the Candida Genome Database.

### qRT-PCR

Total RNA was extracted from cells grown to the logarithmic phase in SC-trp medium at 37 °C using a RNeasy Mini Kit (Qiagen, Hilden, Germany) according to the manufacturer’s instructions. cDNA was synthesized from 2.5 μg of total RNA using a QuantiTect Reverse Transcription kit (Qiagen) in a final volume of 50 μL, and 3 μL of cDNA was used as the template for PCR, which was performed using the QuantiTect SYBR Green PCR kit (Qiagen). qRT-PCR was performed in triplicate using a 7500 Real-Time PCR System (Applied Biosystems, Foster City, CA). The mRNA abundance of the target genes was normalized to that of 18S rRNA. The qRT-PCR was repeated at least three times on independent occasions.

### Western blotting

*C. glabrata* cells were lysed and separated by SDS-PAGE and Phos-tag SDS-PAGE. Both gels were transferred to polyvinylidene difluoride membranes and reacted with the anti-FLAG M2 monoclonal antibody and anti-mouse-IgG horseradish peroxidase. Detailed information on materials and methods for western blotting is presented in the supplementary information file.

## Supplementary information

Supplementary Information.

Supplementary Information 2.

## Data Availability

All data supporting the findings of this study are included in this article and its supplementary information files.
