# Supplementary material for: Roles of Elm1 in antifungal susceptibility and virulence in Candida glabrata
Source: Sci Rep. 2020 Jun 17;10:9789. doi: 10.1038/s41598-020-66620-7 (PMC7299981; doi:10.1038/s41598-020-66620-7)
Supplement: Supplementary file 2 — Supplementary Information 2. [file 41598_2020_66620_MOESM2_ESM.pdf]

Supplementary Information

Roles of Elm1 in antifungal susceptibility and virulence in *Candida glabrata*

Yuya Ito[1), 2)], Taiga Miyazaki[2), 3) *], Yutaka Tanaka[4)], Takashi Suematsu[5)], Hironobu Nakayama[6)], Akihiro Morita[6)], Tatsuro Hirayama[2)], Masato Tashiro[3)], Takahiro Takazono[2), 3)], Tomomi Saijo[2)], Shintaro Shimamura[2)], Kazuko Yamamoto[2)], Yoshifumi Imamura[2)], Koichi Izumikawa[3)], Katsunori Yanagihara[7)], Shigeru Kohno[2)], Hiroshi Mukae[1), 2)]

1) Department of Respiratory Medicine, Nagasaki University Graduate School of Biomedical Sciences, Nagasaki, Japan

2) Department of Respiratory Medicine, Nagasaki University Hospital, Nagasaki, Japan

3) Department of Infectious Diseases, Nagasaki University Graduate School of Biomedical Sciences, Nagasaki, Japan

4) Department of Infection and Host Defense, Tohoku Medical and Pharmaceutical University, Sendai, Japan

5) Central Electron Microscope Laboratory, Nagasaki University Graduate School of Biomedical Sciences, Nagasaki, Japan

6) Faculty of Pharmaceutical Sciences, Suzuka University of Medical Science, Suzuka, Japan

7) Department of Laboratory Medicine, Nagasaki University Hospital, Nagasaki, Japan

*Corresponding: Taiga Miyazaki. e-mail: taiga-m@nagasaki-u.ac.jp

Supplementary materials and methods

Strains, media, cell lines and compounds

The *C. glabrata* strains used in this study are listed in Table S2. *C. glabrata* cells were routinely cultured at 30°C in YPD medium (1% yeast extract, 2% peptone, and 2% dextrose) (Difco Laboratories, Detroit, MI) or SC-trp medium (2% dextrose, 0.67% yeast nitrogen base without amino acids, and 0.19% yeast synthetic drop-out medium supplements without tryptophan). The human type II alveolar epithelial cell line A549 (The American Type Culture Collection, Manassas, VA), was cultured at 37°C in 5% CO_2 in Dulbecco's modified Eagle medium (DMEM; Sigma-Aldrich, St. Louis, MO), supplemented with 10% foetal bovine serum (FBS; Life Technologies, Tokyo, Japan) and 1% penicillin and streptomycin (Sigma-Aldrich). The colorectal adenocarcinoma cell line Caco-2 (The American Type Culture Collection) was cultured in DMEM supplemented with 20% FBS and 1% penicillin and streptomycin.

Micafungin and caspofungin were kindly provided by Astellas (Tokyo, Japan) and Merck & Co., Inc (Kenilworth, NJ, USA), respectively. Congo red and Calcofluor white were purchased from Sigma-Aldrich. Calcium chloride, sodium chloride, SDS and

sorbitol were purchased from Wako Pure Chemical (Osaka, Japan). All compounds were dissolved in distilled water prior to used.

Plasmid and strain construction

The sequence information of *C. glabrata* genes was obtained from the Candida Genome Database (http://www.candidagenome.org/). The primers and plasmids used in this study are listed in Tables S3 and S4, respectively. All plasmids constructed were verified by PCR and sequencing before use. Transformation was performed using a lithium acetate method [1]. The *C. glabrata elm1Δ* strain was constructed using a one-step PCR–based technique as described previously [2,3]. Briefly, a 1-kb DNA fragment containing *C. glabrata HIS3* was amplified from pBSK-HIS3 using the primer pair (CgELM1-KOF and CgELM1-KOR) tagged with the 57-bp sequences homologous to the flanking regions of the *ELM1* open reading frame (ORF). *C. glabrata* strain KUE200 was transformed with the deletion construct, and the resulting transformants were selected by histidine prototrophy. PCR and sequencing were performed to verify that the desired homologous recombination occurred at the target locus.

For the construction of a *C. glabrata elm1Δ* + *ELM1* complemented strain, the intact *ELM1* gene was reintroduced into the *C. glabrata elm1Δ* strain. Briefly, a 1-kb DNA fragment containing *C. glabrata TRP1* was amplified from p916TRP1 using the primer pair (CgELM1-TRP1-F and CgELM1-TRP1-R) tagged with the 66-bp sequences homologous to the 3' flanking region of *ELM1*[4]. *C. glabrata* strain KUE200 was transformed with the PCR construct, and the resulting transformants were selected by tryptophan prototrophy. Subsequently, a 3.0-kb PCR fragment containing the *ELM1*-*TRP1* region was amplified from the genome of this strain using the primer pair ELM1-CHF and ELM1-CHR. The *elm1Δ* strain (TG352) was transformed with the PCR construct, and the resulting transformants were selected by tryptophan prototrophy and verified by PCR and sequencing.

To generate an overexpression plasmid, the 1833-bp *C. glabrata ELM1* ORF was amplified from the genomic DNA of CBS138 using the primer pair (CgELM1-F-BamHI and CgELM1-R-SalI) tagged with 15-bp sequences homologous to the *Bam*HI and *Sal*I sites of pCgACTP. The PCR product obtained was inserted between the *Bam*HI and *Sal*I sites of pCgACTP using the In-fusion HD cloning kit (Takara Bio Inc., Shiga,

Japan). pCgACTP-ELM1 was used as the template to generate pCgACTP-ELM1KD, which was generated by deleting from 265 to 277 bases encoding the kinase domain of *C. glabrata ELM1* using the KOD-Plus-mutagenesis kit (Toyobo, Osaka, Japan) and primer pairs CgELM1 793-R and CgELM1 832-F.

To generate pCgACTP-ELM1-3×FLAG and pCgACTP-ELM1KD-3×FLAG, which C-terminally 3×FLAG-tagged pCgACTP-ELM1 and pCgACTP-ELM1KD, respectively, CgELM1-3×FLAG and CgELM1KD-3×FLAG were amplified from the pBSK-ELM1-3×FLAG-TRP1 and pBSK-ELM1KD-3×FLAG-TRP1 by using the primer pair CgELM1-F-BamHI and CgELM1-3×FLAG-SalI. The PCR products obtained were inserted between the *Bam*HI and *Sal*I sites of pCgACTP using the In-fusion HD cloning kit (Takara Bio). *C. glabrata* strain (TG351) was transformed with pCgACTP, pCgACTP-ELM1, pCgACTP-ELM1KD, pCgACTP-ELM1-3×FLAG, and pCgACTP-ELM1KD-3×FLAG, and the resulting transformants were selected by tryptophan prototrophy and verified by sequencing and qRT-PCR. In the qRT-PCR assay, the *ELM1* overexpression stain (TG353) exhibited approximately 12-fold increase in *ELM1* expression relative to the wild-type strain.

Virulence assay using a mouse mode of disseminated candidiasis

Logarithmic phase *C. glabrata* cells grown in SC-trp medium at 37°C were washed and adjusted to 1.0×10^7 cells/mL in sterile saline. The actual CFU were confirmed by plating serial dilutions of the cell suspensions on YPD plates. Groups of 9 female, 8-week-old, specific-pathogen-free BALB/c mice (Charles River Laboratories Japan Inc., Atsugi, Japan) were injected with 0.2 mL of *C. glabrata* cell suspension via the lateral tail vein. All mice were housed in a pathogen-free environment in groups of four or five in filter-top cages with access to food and water *ad libitum*. Mice were euthanized at 7-days post-injection, and the spleen, lung, and bilateral kidneys were excised. The organs were homogenized, diluted in sterile saline, and plated on YPD agar plates. Colonies were counted after a 48 h of incubation at 30°C, and CFUs per organ were calculated. Statistical analyses were performed using the Kruskal-Wallis test with Dunn's multiple comparison post-test using GraphPad Prism 5 software (GraphPad Software, La Jolla, CA). For histopathological analyses, mice were euthanized at 3-days post-injection, and the lung was excised, fixed with 10% formalin, and stained by periodic acid-Schiff (PAS).

Western blotting

Logarithmic phase cells grown in SC-trp medium at 30°C were lysed using a Minute Total Protein Extraction Kit for Microbes with Thick Cell Walls (Invent Biotechnologies, Inc., Eden Prairie, MN) according to the manufacturer's instructions. Lysates were separated by SDS-PAGE (Thermo Fisher Scientific, Inc., Waltham, MA) and Phos-tag SDS-PAGE (Wako Pure Chemical). After electrophoresis, the Phos-tag gel was washed twice with Tris-Glycine buffer containing 5 mM EDTA and once with Tris-Glycine buffer only. Both gels were transferred to polyvinylidene difluoride membranes (Bio-Rad Laboratories, Inc., Hercules, CA) and reacted with an anti-FLAG M2 monoclonal antibody (Sigma-Aldrich) and anti-mouse-IgG horseradish peroxidase (GE Healthcare, Chicago, IL). After sequential washes with Tris-buffered saline (TBS) with Tween-20 and TBS, each protein was visualized using SuperSignal West Femto Maximum Sensitivity Substrate (Thermo Fisher Scientific, Inc.) and a ChemiDoc Touch Imaging system (Bio-Rad Laboratories, Inc.).

Supplementary Figure Legends

Figure S1. Fungal burden in a mouse model of disseminated candidiasis and histopathological examination of the lungs.

(a) Groups of nine immunocompetent mice were intravenously inoculated with 2.0×10^6 cells of each *C. glabrata* strain and sacrificed at 7-days post-inoculation, followed by determination of CFU/organ in specific organs. The geometric mean is shown as a bar. Representative data from two independent experiments are shown. Statistical analyses were performed and figures were created by using GraphPad Prism 5 software (GraphPad Software, La Jolla, CA) (https://www.graphpad.com/scientific-software/prism/). *C. glabrata* strains: WT, TG11; *elm1*Δ, TG352; and *ELM1*-overexpression, TG353. *$P < 0.05$, Kruskal–Wallis test with Dunn's post-test. (b) Histopathologic examination performed at 3-days post-injection. The excised lung was fixed with 10% formalin and PAS stained. Photographs are representative of two independent examinations. Magnification, 400×.

Figure S2. Cell morphology, growth curves, and spot dilution assays of the *ELM1*-

reconstituted and *ELM1*-KD strains.

(a) Logarithmic phase *C. glabrata* cells grown in SC-trp medium at 30℃ were stained with Calcofluor white. Stained cells were observed by microscopy using bright-field and BZ-X filter for DAPI. *C. glabrata* strains: *elm1*Δ+*ELM1*, TG354; and *ELM1*-KD, TG355. Scale bars, 20 µm. (b) Logarithmic phase *C. glabrata* cells grown in SC-trp medium at 37°C were washed twice with dH_2O, diluted to an OD_{600} of 0.1 with fresh SC-trp medium and incubated at 37°C with shaking at 200 rpm. The OD_{600} of cultures was measured at 2, 4, 6, 8, 10, 12, 24 and 30 h. *C. glabrata* strains: *elm1*Δ+*ELM1*, TG354; and *ELM1*-KD, TG355. Error bars represent standard deviations. The average doubling times per strain were obtained from three independent experiments: *elm1*Δ+*ELM1*, 1.43 h; and *ELM1*-KD, 2.11 h. (c) Serial 10-fold dilutions of logarithmic phase *C. glabrata* cells were spotted onto SC-trp agar plates containing the indicated compounds at the specified concentrations and incubated at 30°C (unless otherwise specified) for 48 h and photographed. Images are representative of three independent replicate experiments. *C. glabrata* strains: *elm1*Δ+*ELM1*, TG354; and *ELM1*-KD, TG355.

Figure S3. Sequence analysis of *C. glabrata* Elm1.

(a) Schematic representation of the kinase domains of *C. glabrata* and *S. cerevisiae* Elm1. The red area indicates the kinase domain. (b) Sequence alignment of the kinase domains of *C. glabrata* and *S. cerevisiae* Elm1. The predicted kinase domain is underlined.

Figure S4. Western blot analysis.

Logarithmic phase cells grown in SC-trp medium at 30°C were lysed and separated by SDS-PAGE and Phos-tag SDS-PAGE. Both gels were transferred to polyvinylidene difluoride membranes and reacted with the anti-FLAG M2 monoclonal antibody and anti-mouse-IgG horseradish peroxidase. The left images show SDS-PAGE and the right images show Phos-tag SDS-PAGE. The original full-length images are shown in lower panels. *C. glabrata* strains: *elm1Δ* + ELM1-3×FLAG, TG355; *elm1Δ* + ELM1KD-3×FLAG, TG356; WT control 1, TG357; and WT control 2, CBS138. Marker: Precise Plus Protein WesternC Standards (BIO-RAD) for SDS-PAGE and WIDE-VIEW Prestained Protein Size Marker (Wako) for Phos-tag SDS-PAGE.

Figure S5. Schematic representation of the downstream effectors regulated by Elm1 in *S. cerevisiae*.

S. cerevisiae Elm1 is involved in multiple cellular functions, including cytokinesis, carbon metabolism, morphogenesis, by phosphorylating the downstream proteins (see the Discussion section for details).

Table S1. List of genes upregulated in the *C. glabrata elm1Δ* strain (Excel)

Table S2. List of genes downregulated in the *C. glabrata elm1Δ* strain (Excel)

Table S3. List of genes upregulated in the *C. glabrata ELM1*-KD strain (Excel)

Table S4. List of genes downregulated in the *C. glabrata ELM1*-KD strain (Excel)

Table S5. Altered expression levels of adhesin-related and chitin-related genes in the *C. glabrata ELM1*-KD strain

**Systematic name and function**	**Gene**	**Fold change in expression**	***S. cerevisiae* ortholog**
Upregulated adhesin-related gene			
CAGL0M00110g	*AWP11*	6.9	
CAGL0I11011g		6.6	
CAGL0J00110g		6.5	
CAGL0L00157g		5.3	
CAGL0I00220g	*EPA23*	4.9	*FLO1*
CAGL0H00110g		4.7	
CAGL0E00110g		4.0	
CAGL0F09273g		4.0	
CAGL0I00110g		3.9	
CAGL0J11891g	*AWP3*	3.9	
CAGL0A00110g	*EPA19*	3.8	*FLO1*
CAGL0H10626g	*AWP13*	3.6	
CAGL0H00132g		3.4	
CAGL0G10219g	*AWP12*	3.3	
CAGL0F00110g	*AWP10*	3.3	
CAGL0G10175g	*AWP6*	3.1	*DAN1*
CAGL0K13002g	*AED2*	2.7	
CAGL0C03575g		2.5	*AGA1/YNR044W*
CAGL0J02530g		2.1	
CAGL0E00275g	*EPA20*	1.9	FLO5
CAGL0B05093g	*AWP9*	1.8	
CAGL0K10164g		1.6	*SED1/YDR077W*
CAGL0G04125g		1.5	*SAG1*
CAGL0I10098g	*PWP7*	1.4	*FLO5*
CAGL0K13024g	*AED1*	1.4	

CAGL0J00253g		1.3	*MTL1/YGR023W*
CAGL0A04873g		1.2	
CAGL0J02508g	*AWP1*	1	
CAGL0M00132g	*EPA12*	1	*FLO1*
Downregulated adhesin-related gene			
CAGL0L13332g	*EPA13*	-3.5	*FLO9*
CAGL0B00110g	*AWP8*	-2.7	
CAGL0E02915g		-2.0	*SCW11/YGL028C*
CAGL0I10340g	*PWP5*	-1.5	*FLO1*
CAGL0J04950g	*EPA21*	-1.3	*FLO1*
Upregulated chitin-related gene			
CAGL0I04840g	*CHS3B*	1.5	*CHS3*
Downregulated chitin-related gene			
CAGL0M09779g	*CTS1*	-3.2	*CTS1*

Table S6. *C. glabrata* strains used in this study

Strain	Genotype or description	Reference or source
CBS138	ATCC2001 (*C. glabrata* wild-type)	[5]
2001T	*trp1Δ* (a derivative of CBS138)	[6]
KUE200	*his3Δ, trp1Δ*	[7]
TG11	2001T containing pCgACTP	[8]
TG351	*elm1Δ*::HIS3, *trp1*Δ (made from KUE200)	This study
TG352	TG351 containing pCgACTP	This study
TG353	TG351 containing pCgACTP-ELM1	This study
TG354	*his3*::*ELM1-TRP1* (made from TG351)	This study
TG355	TG351 containing pCgACTP-ELM1KD	This study
TG356	TG351 containing pCgACTP-ELM1-	This study

	3×FLAG	
TG357	TG351 containing pCgACTP-ELM1KD-3×FLAG	This study
TG358	2001T containing pCgACTP-ELM1-3×FLAG	This study

Table S7. Primers used in this study

For gene deletion	Sequence (5' to 3')
CgELM1-KOF	AAGAGAACATTACTTGAAAAGAAATTAAGTATACTGCAAAGAATTGAAGAAATAGTGGCCGCTGATCACG
CgELM1-KOR	TAATAAAACAAACTGGTCTTGCTATTTCTTGGTTTAAGTTTGCGATACAGTTATTACATCGTGAGGCTGG
For gene cloning	Sequence (5' to 3')
CgELM1-F-BamHI	TAGAACTAGTGGATCCATGTCTGACAATGAATACCCTACAG
CgELM1-R-SalI	TGTTCTCGAGGTCGACTTAGACCTCAGCTGAATCTGATTC
CgELM1-TRP1-F	TATTACACTACTAGAACAGATAAAGTTATGAATTAGATTGCTTTTGAATGAAATTTCATATTATGCATCGTGAGGCTGG
CgELM1-TRP1-R	ATCCAACGATACAGTTAGAAACATAAACTCGTATTTAGAATCAGATTCAGCTGAGGTCTAATAACTGGCCGCTGATCACG
CgELM1-CHF	AAACTAATTGACCCTCTCTGC
CgELM1-CHR	CGTCTGTTACAGATTCGGAC
CgELM1 793-R	ACCATTAATCTTCATAAATTTCAA
CgELM1 832-F	CAGCAATATATTGGAAGGTT
CgELM1-3×FLAG-R-SalI	TGTTCTCGAGGTCGACTTACTTGTCATCGTCATCCTTG

Table S8. Plasmids used in this study

Plasmid	Description	Reference of source
pBSK-HIS	pBluescriptII SK$^+$ containing *C. glabrata HIS3* at the *Xho*I site	[8]
p916TRP1	pBluescriptII SK$^+$ containing *C. glabrata TRP1* at the *Not*I-*Sal*I site	This study
pBSK-ELM1-3×FLAG-TRP1	pBluescriptII SK$^+$ containing *C. glabrata ELM1*-3×FLAG-*TRP1*-*ELM1* 3'UTR at the *Bam*HI-*Kpn*I site	This study
pBSK-ELM1KD-3×FLAG-TRP1	pBluescriptII SK$^+$ containing *C. glabrata ELM1c.265-277del*-3×FLAG-*TRP1*-*ELM1* 3'UTR at the *Bam*HI-*Kpn*I site	This study
pCgACTP	*S. cerevisiae PGK1* promoter and *C. glabrata HIS3* 3'UTR were inserted into the *Sac*I-*Kpn*I site of pCgACT	[8]
pCgACTP-ELM1	*C. glabrata ELM1* ORF was inserted into the *Bam*HI-*Sal*I site of pCgACTP	This study
pCgACTP-ELM1KD	*C. glabrata ELM1 c.265_277del* was inserted into the *Bam*HI-*Sal*I site of pCgACTP	This study
pCgACTP-ELM1-3×FLAG	*C. glabrata ELM1* ORF- 3×FLAG was inserted into the *Bam*HI-*Sal*I site of pCgACTP	This study
pCgACTP-ELM1KD-3×FLAG	*C. glabrata ELM1* ORF*c.265_277del*-3×FLAG was inserted into the *Bam*HI-*Sal*I site of pCgACTP	This study

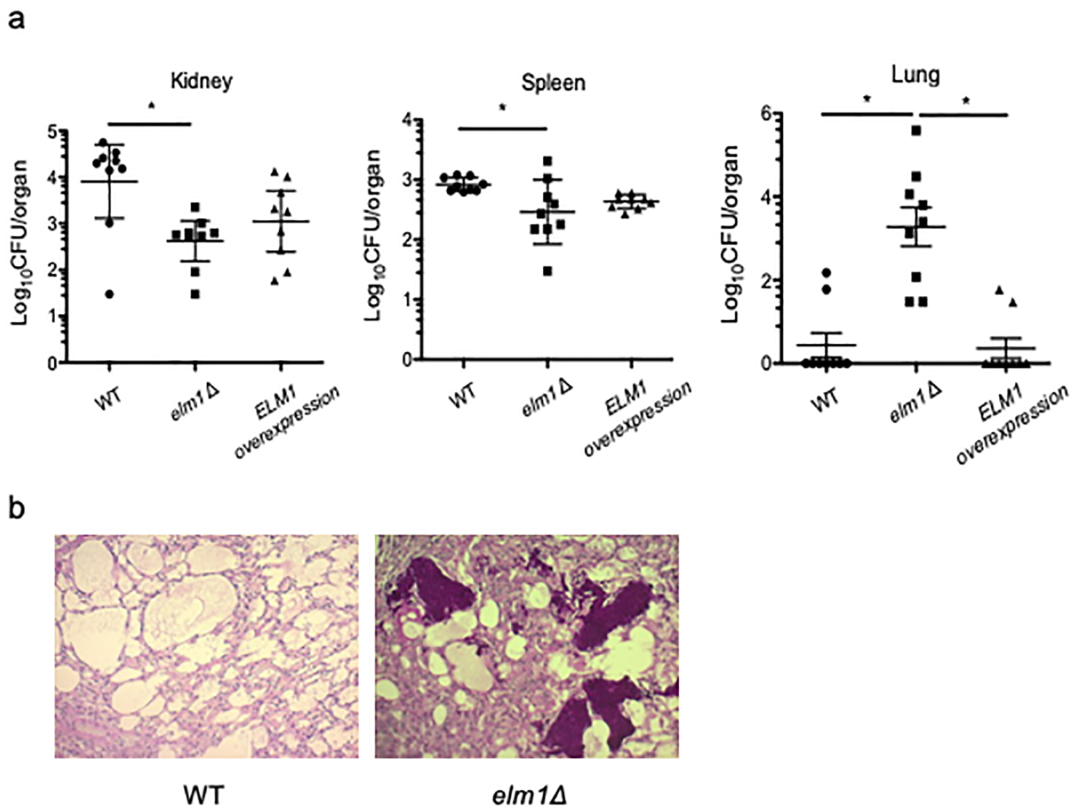

Figure S1. Fungal burden in a mouse model of disseminated candidiasis and histopathological examination of the lungs.

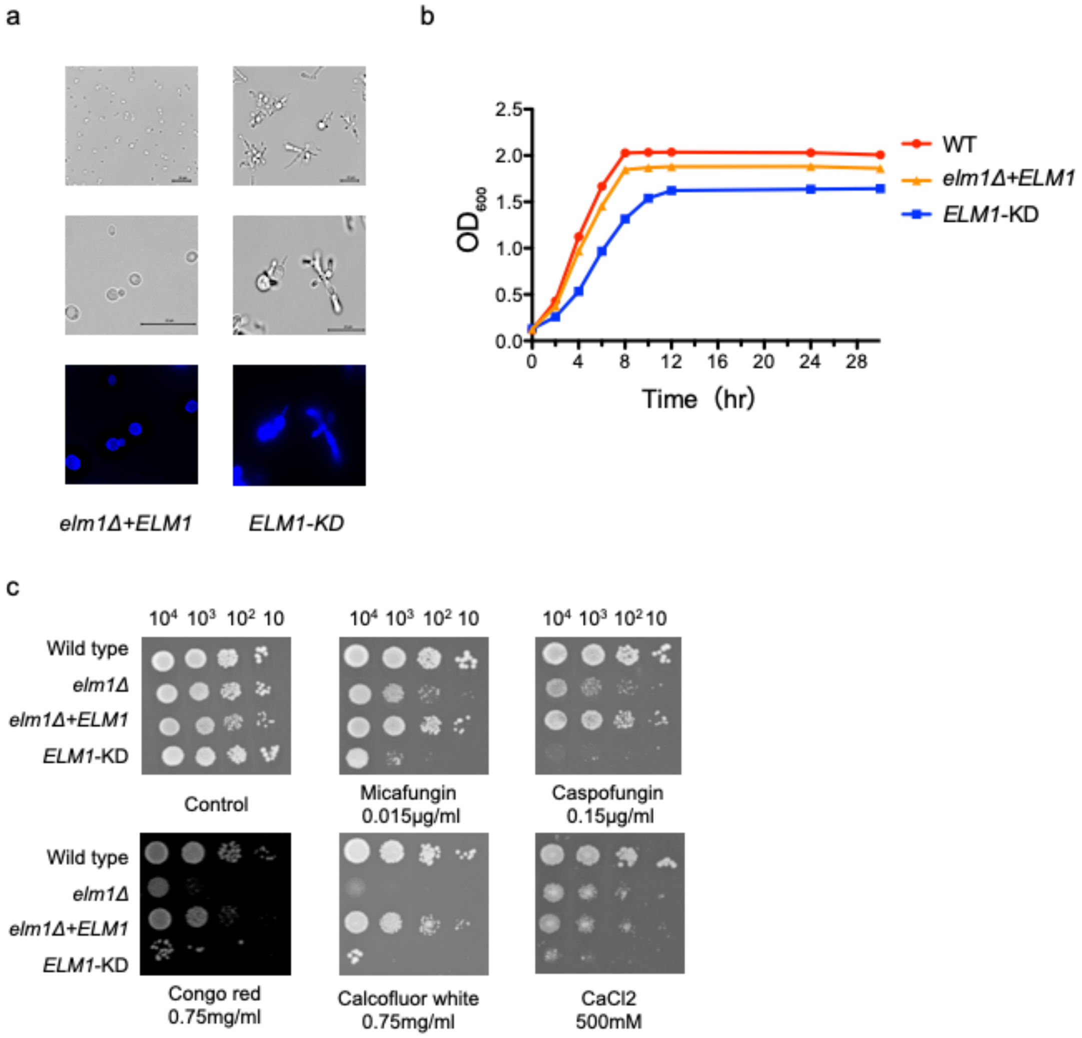

Figure S2. Cell morphology, growth curves, and spot dilution assay of the *ELM1*-reconstituted and *ELM1*-KD strains.

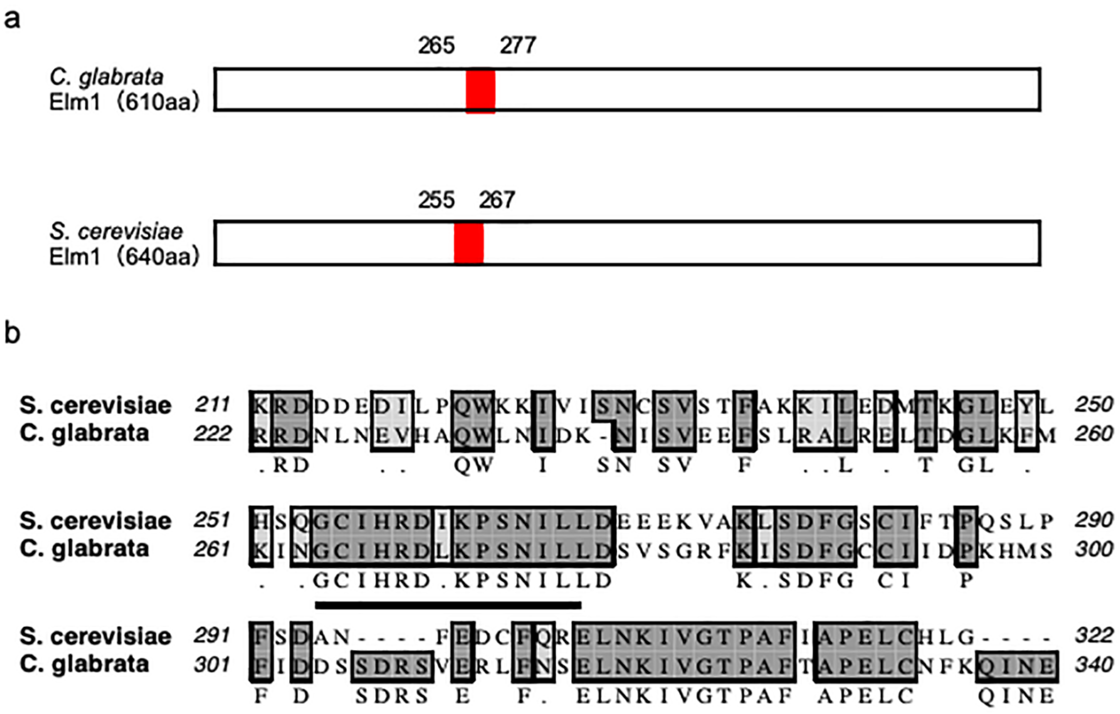

Figure S3. Sequence analysis of *C. glabrata* Elm1.

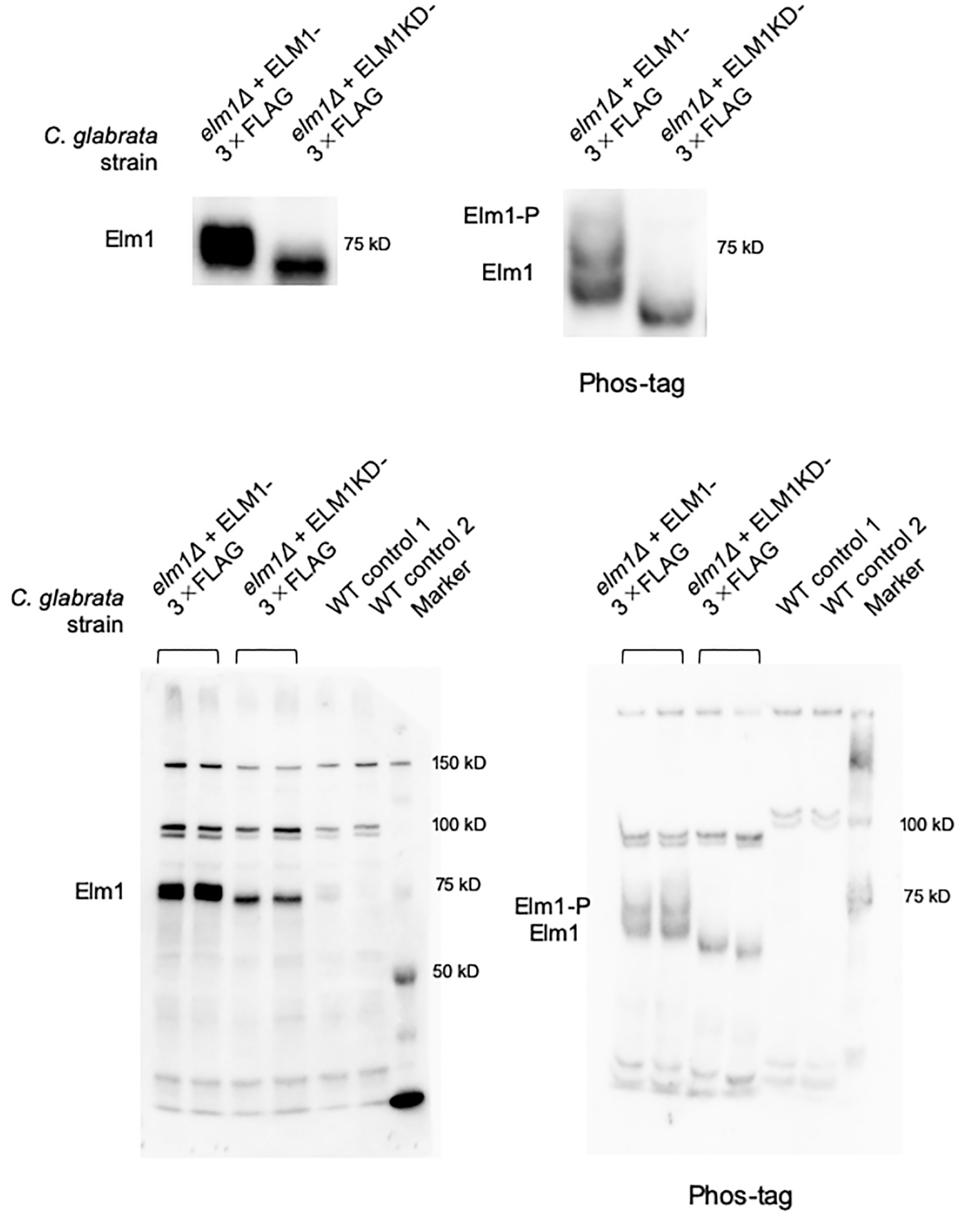

Figure S4. Western blot analysis.

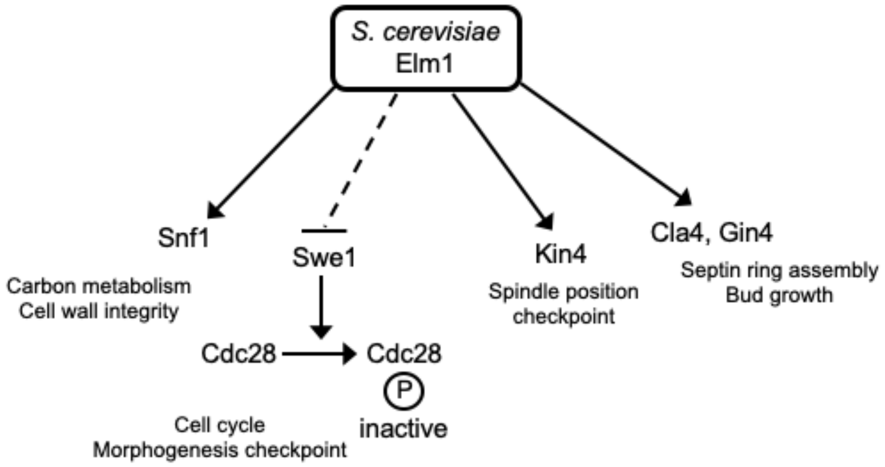

Figure S5. Schematic diagram of the phosphorylate pathway of *S. cerevisiae*.

References

1 Falkow, B. P. C. a. S. Efficient Homologous and Illegitimate Recombination in the Opportunistic Yeast Pathogen Candida glabrata. *Genetics* **151**, 979-987 (1999).

2 Gola, S., Martin, R., Walther, A., Dünkler, A. & Wendland, J. New modules for PCR-based gene targeting inCandida albicans: rapid and efficient gene targeting using 100 bp of flanking homology region. *Yeast* **20**, 1339-1347, doi:10.1002/yea.1044 (2003).

3 Cota, J. M. *et al.* Increases in SLT2 expression and chitin content are associated with incomplete killing of Candida glabrata by caspofungin. *Antimicrob Agents Chemother* **52**, 1144-1146, doi:10.1128/AAC.01542-07 (2008).

4 Inukai, T. *et al.* The mannoprotein TIR3 (CAGL0C03872g) is required for sterol uptake in Candida glabrata. *Biochim Biophys Acta* **1851**, 141-151, doi:10.1016/j.bbalip.2014.11.002 (2015).

5 Dujon B, S. D., Fischer G et al. . Genome evolution in yeasts. *Nature* **430**, 35-44 (2004).

6 Kunio Kitada, E. Y. a. M. A. Cloning of the Candida glabrata TRP1 and HIS3 genes, and construction of their disruptant strains by sequential integrative transformation. *Gene* **165**, 203-206 (1995).

7 Ueno, K. *et al.* Development of a highly efficient gene targeting system induced by transient repression of YKU80 expression in Candida glabrata. *Eukaryot Cell* **6**, 1239-1247, doi:10.1128/EC.00414-06 (2007).

8 Miyazaki, T. *et al.* Roles of calcineurin and Crz1 in antifungal susceptibility and virulence of Candida glabrata. *Antimicrob Agents Chemother* **54**, 1639-1643, doi:10.1128/AAC.01364-09 (2010).
